# Early and individualized goal-directed therapy for trauma-induced coagulopathy

**DOI:** 10.1186/1757-7241-20-15

**Published:** 2012-02-24

**Authors:** Herbert Schöchl, Marc Maegele, Cristina Solomon, Klaus Görlinger, Wolfgang Voelckel

**Affiliations:** 1 Ludwig Boltzmann Institute of Experimental and Clinical Traumatology, Vienna, Austria; 2 Department of Anaesthesiology and Intensive Care Medicine, AUVA Trauma Centre, Salzburg, Austria; 3 Department of Trauma and Orthopaedic Surgery, University of Witten/Herdecke, Cologne-Merheim Medical Centre (CMMC), Cologne, Germany; 4 Department of Anaesthesiology and Intensive Care Medicine, University Hospital of Essen, Essen, Germany

**Keywords:** ROTEM, TEG, trauma, goal-directed coagulation therapy

## Abstract

Severe trauma-related bleeding is associated with high mortality. Standard coagulation tests provide limited information on the underlying coagulation disorder. Whole-blood viscoelastic tests such as rotational thromboelastometry or thrombelastography offer a more comprehensive insight into the coagulation process in trauma. The results are available within minutes and they provide information about the initiation of coagulation, the speed of clot formation, and the quality and stability of the clot. Viscoelastic tests have the potential to guide coagulation therapy according to the actual needs of each patient, reducing the risks of over- or under-transfusion. The concept of early, individualized and goal-directed therapy is explored in this review and the AUVA Trauma Hospital algorithm for managing trauma-induced coagulopathy is presented.

## Introduction

Major brain injury and uncontrolled blood loss remain the primary causes of early trauma-related mortality [1-3]. One-quarter to one-third of trauma patients exhibit trauma-induced coagulopathy (TIC) [4,5], which is associated with increased rates of massive transfusion (MT) and multiple organ failure (MOF), prolonged intensive care unit and hospital stays, and a four-fold increase in mortality [4]. Most patients with coagulopathy also have uncontrolled bleeding, and early diagnosis of the underlying coagulation disorder is paramount for effective treatment.

One major challenge in treating severely bleeding trauma patients is to determine whether the blood loss is attributable to surgical causes or coagulopathy. If the patient is coagulopathic, it is paramount to characterize the cause of the coagulopathy and whether thrombin generation is impaired or clot quality or stability is diminished. Recent data suggest that whole-blood viscoelastic tests, such as thromboelastometry (ROTEM^®^, Tem International GmbH, Munich, Germany) or thrombelastography (TEG^®^, Haemonetics Corp., Braintree, MA, USA) portray trauma induced coagulopathy (TIC) more accurately and substantially faster than standard coagulation tests [6-8]. There is increasing evidence that these coagulation monitoring devices are helpful in guiding coagulation therapy for heavily bleeding trauma patients according to their actual needs [9].

The intention of this review is to examine the concept of individualized, early, goal-directed therapy for TIC, using viscoelastic tests and targeted coagulation therapy. In addition, the AUVA Trauma Hospital algorithm for managing TIC is presented.

### Value of standard coagulation tests

Fast, reliable diagnosis and characterization of TIC is important. Standard coagulation tests (e.g. prothrombin time [PT], international normalized ratio [INR], prothrombin time index [PTI] and activated partial thromboplastin time [aPTT]) fail to accurately describe the complex nature of TIC for several reasons [4,5]. In vivo coagulation occurs primarily on the surface of platelets and tissue factor-bearing cells [10], and red blood cells (RBCs) also play a significant role in haemostasis [11]. Standard coagulation tests are performed using plasma in the absence of blood cells (these are removed by centrifugation). Also, these tests are stopped upon formation of the first fibrin strands, when only ~5% of the total thrombin has been generated [12]. Moreover, these tests do not assess the quality/the strength of the clot. Hyperfibrinolysis is recognized as a potential contributor to mortality in trauma [13-15], and this aspect is not assessed by standard coagulation tests [16].

Coagulation factors do not decrease homogeneously in severe bleeding. Although there may be a tendency towards excessive thrombin generation, coagulation factor levels are decreased and fibrinogen appears to reach critical levels at an early stage [17-19]. Therefore, measurement of fibrinogen concentration is strongly recommended in trauma patients [14,19]. However, when using artificial colloids, falsely high fibrinogen levels are recorded by some coagulation analysers that employ the Clauss method [20,21]. Artificial colloids also impair fibrin polymerization, and standard laboratory measurement does not represent fibrinogen functionality [22].

Another shortcoming of standard coagulation tests, including fibrinogen concentration measurement, is that the results are available only after a substantial time delay. Median turnaround times of 78-88 minutes have been reported [23,24].

In summary, standard coagulation tests are unable to characterize the complex nature of TIC. They are time-consuming and offer little prognostic value regarding transfusion requirements [25].

### Role of thromboelastometry/thrombelastography

Trauma care providers are increasingly aware that viscoelastic coagulation monitors such as thromboelastometry (ROTEM) and thrombelastography (TEG) are valuable alternatives to standard coagulation testing, providing a more comprehensive overview of the coagulation process [6,8,23,26-29]. In contrast to most standard coagulation measurements, ROTEM and TEG can be used as point-of-care methods. Viscoelastic tests are performed in whole blood as opposed to plasma, which provides a better reflection of the *in vivo *situation, avoids the need for centrifugation and allows initial results to be available within minutes [23,30].

**Figure 1:**
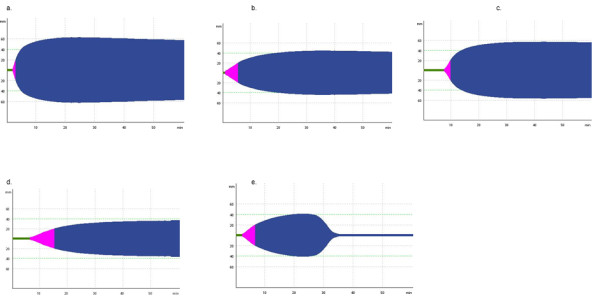
**Examples of ROTEM traces using the EXTEM test: **a. normal test result. b. reduced maximum clot firmness (MCF). c. delayed initiation of coagulation (prolonged coagulation time [CT]). d. prolonged CT and reduced MCF. e. hyperfibrinolysis.

ROTEM and TEG tests provide dynamic information on the speed of coagulation initiation, kinetics of clot growth, clot strength, and breakdown of the clot [16,31]. The ROTEM device uses a plastic pin immersed vertically into a cup containing the blood sample; the pin is rotated slowly, backwards and forwards, through an angle of 4.75°. The device has four channels, allowing four tests to be performed simultaneously. Two basic ROTEM tests that use intrinsic activation (INTEM) and extrinsic activation (EXTEM) provide information on the general coagulation status (impaired, normal, and hypercoagulable). Figure 1 shows examples of EXTEM plots with normal and impaired coagulation. A set of standard reagents can additionally be used to characterize coagulopathy. For example, the FIBTEM test, which comprises the EXTEM assay with added cytochalasin D to inhibit platelets, provides information on the fibrin component of the clot.

The TEG device uses a stationary torsion wire and the cup is rotated. TEG is a two channel system that uses either kaolin as an activator or a combination of kaolin and tissue factor ('rapid TEG' or 'r-TEG' assay). The functional fibrinogen test is equivalent to the ROTEM FIBTEM test. These methods and devices are described in detail elsewhere [16,31].

### Assessment of platelet function

Platelet count provides quantitative information on platelet numbers, but it provides no information on platelet function. Prior treatment with platelet inhibitors such as aspirin or thienopyridines is commonly encountered among emergency room patients and, although platelet function may potentially influence patient outcomes [32], platelet inhibitor therapy cannot be assessed adequately by standard viscoelastic analyses [33]. POC monitoring devices allowing rapid assessment of platelet function (e.g. Multiplate^®^; Roche Diagnostics, Munich, Germany) have been developed recently [34]. Whole blood is added to test cells which incorporate two independent sensor units. Platelet aggregation takes place on the sensor units following addition of a platelet activator. Different platelet activators are used such as adenosine diphosphate (ADPtest), arachidonic acid (ASPItest), and thrombin receptor-activating peptide-6 (TRAP-6; TRAPtest). The platelets' ability to adhere to the metal sensors and build aggregates is measured by the electrical resistance change between two sensor wires. The impedance change between each pair of wires is recorded as an aggregation curve and expressed in "aggregation units" (U). In the AUVA trauma hospital, Mulitplate was used as a diagnostic tool for assessing platelet function in all patients admitted to the ER between 2009 and 2011 [32]. In light of our data published in 2011, [32] we now use Multiplate only in patients who are unconscious (meaning that anamnesis is impossible) or known to be taking platelet inhibitor medication.

### Rationale for massive transfusion protocols

It has been shown that the majority of massively transfused patients who exsanguinated within the first 24 hours after hospital admission received insufficient amounts of fresh frozen plasma (FFP) and platelet concentrate (PC) [35]. Inadequate replacement of coagulation factors during the initial care was identified as the main cause of prolonged INR upon admission to the intensive care unit (ICU) [36]. Recent data indicate that early and aggressive transfusion of FFP and PC is associated with improved survival rates among major trauma patients [37-42]. To increase the effectiveness of coagulation therapy, massive transfusion protocols (MTPs) have been implemented. Predefined sets of RBC concentrate, FFP and PC are issued by the blood bank after the activation of such MTPs [43,44]. However, MTPs vary in format between trauma centres, with different ratios of FFP to RBCs and PC [45]. MT prediction scores have been developed for early identification of at-risk patients, with the aim of initiating MTPs as early as possible thereby minimizing treatment delays. These scores are based on anatomic findings and/or rapidly available laboratory data such as PT and base deficit [46-49].

### What is the optimal ratio of FFP:RBC?

It has long been assumed that high-volume plasma replacement therapy may potentially avoid or correct coagulopathy in severely bleeding patients. A computer model by Hirshberg et al. has indicated that late transfusion of plasma may be insufficient for preventing or correcting coagulopathy [50]. Moreover, the optimum FFP:RBC replacement ratio was calculated in the same study to be approximately 2:3 [50]. Similarly, data from a military centre show that the optimal FFP:RBC ratio for minimizing mortality among trauma patients receiving MT is close to 1:1.4 [37]. A retrospective analysis of civilian trauma patients reported that a high FFP:RBC ratio (> 1:1) significantly lowers intraoperative, 24-hour and 30-day mortality [42]. An abundance of studies support early and aggressive coagulation management with FFP [51]. However, the optimal ratio of FFP:RBC is still under discussion [7,52,53].

For example, Kashuk et al. suggest that the optimal ratio of FFP:RBC appears to be in the range of 1:2 to 1:3 [7], with no improvement in survival rate among patients receiving higher ratios. More recently, Davenport et al. found no improvement in coagulation status when FFP:RBC were transfused in a ratio of 1:1 compared with 1:2 or 3:4 [52]. Simmons et al. reported data from the Iraq War in which a change of clinical practice guidelines toward higher FFP:RBC ratios resulted in significantly higher FFP transfusion but no improvement in survival [54]. Similarly, implementation of an MTP targeting an FFP:RBC ratio of 1:1 in a Scandinavian trauma centre significantly increased transfusion of FFP without improving mortality [55].

### Timing of haemostatic intervention is crucial

There is no doubt that coagulation therapy should be started as early as possible. Riskin et al. reported a significant reduction in mortality from 45% to 19% after implementation of an MTP despite unchanged FFP:RBC ratios. The most likely reason for the improvement was a significant reduction in mean time to administration of FFP, from 254 to 169 minutes [56]. Early haemostatic therapy appears to prevent the development of coagulopathy in some patients, eliminating the need for MT; plasma transfusion seems to be most effective during the first 2-3 hours of care for massively bleeding patients [57].

### FFP: safety and the need for selective use

High-volume FFP transfusion is associated with considerable side effects [55,58-61]. Chaiwat et al. reported a dose-dependent relationship between FFP transfusion and acute respiratory distress syndrome (ARDS) in trauma patients [58]. In another study, FFP transfusion was independently associated with increased risk of MOF and ARDS in patients who survived beyond 48 hours [62].

There is little evidence that patients receiving < 10 units (U) of RBCs benefit from FFP transfusion. Inaba et al. showed that in non-MT trauma patients (< 10 U RBC/24 hours), plasma administration increased complications (e.g. ARDS, pneumonia, sepsis and MOF), without improving survival [59]. Other data show a greater increase in side effects with FFP among patients receiving < 6 U RBCs within the first 6 hours [60]. Recently, Sambasivan et al. analysed outcome data form 1,788 non-MT patients (< 10 U RBC/24 hours) who received FFP and PC. A high ratio of FFP:RBC and PC:RBC was associated with fewer ventilator-free and fewer ICU-free days [61]. Therefore, early identification of patients who are prone to MT seems important.

In summary, FFP transfusion is associated with serious side effects, and transfusion triggers should be chosen carefully. In particular, patients receiving < 10 U RBCs do not appear to benefit from FFP transfusion.

### Concept of early and individualized goal-directed coagulation therapy

**Figure 2:**
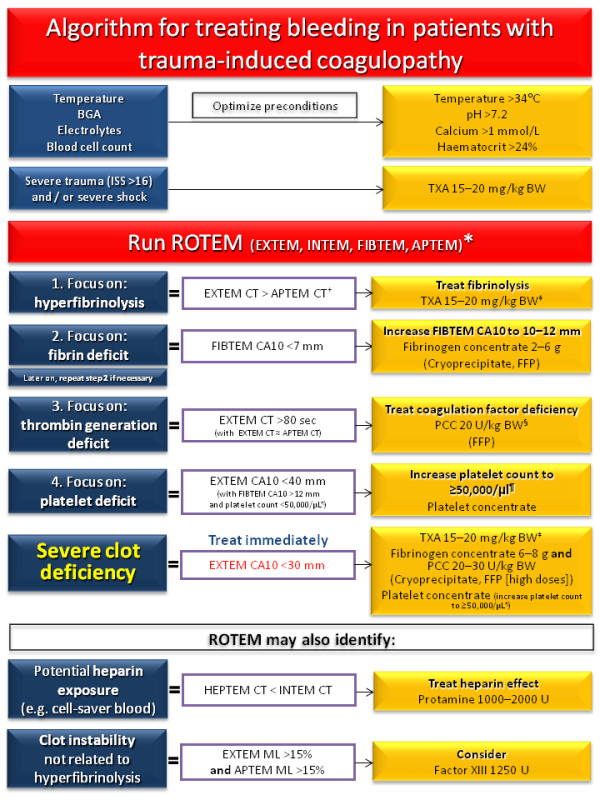
**ROTEM-guided treatment algorithm: managing trauma-induced coagulopathy and diffuse microvascular bleeding (AUVA Trauma Hospital, Salzburg, Austria)**. The algorithm represents standard operating procedure for ROTEM-guided haemostatic therapy upon admission of trauma patients to the emergency room. In parentheses: haemostatic agents suggested for use in clinics where coagulation factor concentrates are not available. * For patients who are unconscious or known to be taking platelet inhibitor medication, Multiplate tests (adenosine diphosphate [ADP] test, arachidonic acid [ASPI] test, and thrombin receptor activating peptide-6 [TRAP] test) are also performed. ^§ ^If decreased ATIII is suspected or known, consider co-administration of ATIII. ^† ^Any major improvement in APTEM parameters compared to corresponding EXTEM parameters may be interpreted as a sign of hyperfibrinolysis. ^‡ ^Only for patients not receiving TXA at an earlier stage of the algorithm. Traumatic brain injury: platelet count 80,000-100,000/μl. Normal values: EXTEM/APTEM coagulation time (CT): 38-79 seconds; EXTEM/APTEM clot amplitude at 10 minutes (CA10): 43-65 mm; EXTEM/APTEM maximum lysis (ML) < 15%; FIBTEM CA10: 7-23 mm; INTEM CT: 100-240 seconds. CA10, clot amplitude at 10 minutes; BGA, blood gas analysis; BW, body weight; Ca, calcium; CT, clotting time; FFP, fresh frozen plasma; ISS, injury severity score; MCF, maximum clot firmness; ML, maximum lysis; PCC, prothrombin complex concentrate; TXA, tranexamic acid.

In contrast to a fixed ratio of FFP:PC:RBC, goal-directed coagulation therapy aims to adapt treatment to the actual needs of the individual patient, based on viscoelastic test results (Figure 2) [6,8]. The short turnaround times of ROTEM and TEG assays allow rapid diagnostic testing and individualized drug therapy to be based on test results, with a feedback loop to monitor and optimize treatment effectiveness and to minimize side-effects. This "theragnostic" concept offers several potential advantages, and these are discussed below.

#### Rapid assessment of coagulation status and prediction of the need for massive transfusion

In severe trauma patients, it is crucial to receive rapid information on the patient's current coagulation status. The first ROTEM or TEG test results are available within minutes [30]. Low maximum clot firmness (MCF) in EXTEM, INTEM and FIBTEM or maximum amplitude (MA, the equivalent TEG parameter) has been identified as an important determinant of RBC transfusion [23,30,63-67]. In one study, MA but not standard coagulation tests (INR, PT, aPTT) was predictive for blood product transfusions [66]. Leemann et al. showed that low INTEM MCF and low haemoglobin levels were independent risk factors for MT [63]. TEG clot strength (G) on admission to the ER has been reported to provide consistent prediction of MT in trauma patients [65]. Davenport et al. observed that ROTEM clotting times in trauma patients show only a trend toward prolongation in coagulopathic patients (116 vs. 66 seconds; p = 0.0068). However, EXTEM clot amplitude < 35 mm at 5 minutes was identified as a predictor of MT with a detection rate of 71% [23]. A retrospective study of major trauma patients (ISS > 16) showed that a low FIBTEM amplitude (< 4 mm) and/or a low EXTEM amplitude (< 35 mm) at 10 minutes (CA10) reliably predicts MT [30].

AUVA TRAUMA HOSPITAL TREATMENT ALGORITHM FOR TIC - DIAGNOSIS

To provide rapid information on the coagulation status of major trauma patients: run **ROTEM tests **(EXTEM, INTEM, FIBTEM and APTEM) to assess clotting times and/or clot quality on admission to the ER. For patients who are unconscious or known to be taking platelet inhibitor medication, **Multiplate tests **(adenosine diphosphate [ADP] test, arachidonic acid [ASPI] test, and thrombin receptor activating peptide-6 [TRAP] test) are also performed, to assess platelet function.

#### Improving and maintaining clot quality in TIC

Based on the evidence available, it appears reasonable to focus goal-directed coagulation therapy on the maintenance or restoration of clot strength. Clot strength is determined by interactions between the fibrin network, activated platelets and activated factor XIII.

### Fibrinogen supplementation

Fibrinogen seems to reach critically low levels very early after trauma [9,13,23,30]. Current European guidelines recommend a plasma fibrinogen concentration in trauma patients of no less than 1.5-2.0 g/L [29]. However, there is limited evidence that fibrinogen administration improves outcomes in trauma patients. A retrospective military study reported increased survival among patients receiving a high fibrinogen:RBC ratio (0.48 g fibrinogen per unit of RBCs), compared with those receiving a lower ratio (0.1 g fibrinogen per unit of RBC) [68].

FFP is one potential source of fibrinogen, but it is collected from healthy volunteers who may occasionally have low fibrinogen levels. Fibrinogen concentrations of ~2 g/L have been found in both FFP and solvent-detergent (SD) plasma [69]. Chowdhury et al. compared changes in coagulation factor levels after FFP transfusion of 12 mL/kg bodyweight or 35 mL/kg bodyweight, and observed a substantial increase in fibrinogen solely in the high-FFP volume group [70]. Prior to infusion, FFP must be thawed and this is a time-consuming process. Pre-thawing of FFP may avoid this delay, but storage time is limited. Lyophilized plasma, which is immediately available in the ER, represents a possible future solution to these problems [71].

Cryoprecipitate, another source of fibrinogen, is still available in the US and the UK, but it has been withdrawn from some European countries in response to significant safety concerns [72]. The fibrinogen content of a unit of cryoprecipitate can vary widely, from 120 to 796 mg [73]. In patients not receiving plasma components within the preceding 2 hours, cryoprecipitate has been reported to produce a mean increase in fibrinogen concentration of ~0.06 g/U [74]. If using cryoprecipitate to increase plasma fibrinogen concentration, European Guidelines recommend transfusion of 15-20 U in a 70-kg adult [29]. Only limited data show improved survival following transfusion of cryoprecipitate in trauma patients [62].

Fibrinogen concentrate is licensed in some European countries for congenital and acquired bleeding. It can be reconstituted easily and quickly using sterile water or saline [75], allowing rapid and controlled dosing. Approximately 3 g of fibrinogen concentrate is required to raise the plasma concentration by 1 g/L in a 70-kg patient [76]. Fibrinogen concentrate can be administered without thawing or cross-matching, significantly reducing time to infusion [8]. In emergency cases, administration of 6 g in 1-2 minutes has been reported [76]. Post-treatment improvements in clot formation and clot strength can be monitored using viscoelastic tests [8,76]. The safety profile of fibrinogen concentrate appears to be favourable with a low risk of thromboembolic events [77-79], although the available data cannot be considered as definitive in this regard.

AUVA TRAUMA HOSPITAL TREATMENT ALGORITHM FOR TIC

FIBTEM CA10 < 7 mm suggests insufficient fibrin clot formation. Fibrinogen concentrate should be administered until a FIBTEM CA10 of 10-12 mm is reached.

### Platelet transfusion

Platelets are important determinants of clot quality and serve as a matrix for coagulation factors [11]. Upon ER admission, platelet count < 150,000/μL has been reported in only 4% of trauma patients with an injury severity score (ISS) of 5 and in 18% of patients with ISS > 45 [80].

In bleeding trauma patients, it is recommended to maintain platelet count > 50,000/μL and in patients with substantial brain injury, a platelet count > 100,000/μL is suggested as optimal [29]. However, the value of platelet transfusion in a fixed, predefined ratio for the management of TIC is currently unclear. The reported improvements in survival associated with platelet transfusion are subject to survival and selection biases similar to those seen with FFP and the efficacy and safety of platelet transfusion in a predetermined ratio has not been established. Holcomb et al. reported improved early and late survival and decreased rates of haemorrhagic deaths in patients receiving a high ratio of PC:RBC. As a potential side effect of increased platelet transfusion, MOF increased as the PC:RBC ratio increased [81]. The MTP published by Dirks et al. resulted in a significant increase in PC transfusion without any improvement in the survival rate [55]. Similar results were reported by Simmons et al., who observed that the introduction of new clinical practice guidelines forcing early platelet transfusion resulted in no survival benefit [54]. Thus, platelet transfusion in a fixed predefined ratio carries the potential for wasting valuable resources and the risk of complications (e.g. transfusion-related acute lung injury, pathogen transmission).

AUVA TRAUMA HOSPITAL TREATMENT ALGORITHM FOR TIC

EXTEM CA10 < 40 mm and FIBTEM CA10 > 12 mm and platelet count < 50,000/μL suggests sufficient fibrin clot formation, but insufficient platelets to produce adequate clot strength. Platelet concentrate transfusion is indicated.

### Improving initiation of the coagulation process

Thrombin generation is not substantially affected in the early stages of TIC [82]. Brohi et al. reported that generation of prothrombin fragment_1-2 _increased with increasing ISS [83]. Thrombin generation in patients with possible TIC (PT > 18 seconds or INR > 1.5) has been reported as threefold higher than in controls (p = 0.01) [17]. The concept of of early, high-dose FFP transfusion with the aim of increasing thrombin generation must be debated in the light of adequate or even increased thrombin generation in trauma patients upon admission to the ER.

Coagulation time (CT) in ROTEM or reaction time (r-time) in TEG serve as surrogate markers for the speed of initiation of coagulation and can be considered as the whole-blood PT or aPTT. Weiss et al. studied PTI, aPTT, EXTEM CT and INTEM CT in a dilution model [84]. A linear relationship was observed between either PTI or aPTT and the concentration of coagulation factors. However, EXTEM CT remained < 80 seconds (normal range) until coagulation factor activity fell below ~35% of normal.

Therefore, in the AUVA treatment algorithm, EXTEM CT > 80 seconds and INTEM CT > 240 seconds indicate a need for treatment to improve thrombin generation (Figure 2).

Current options for increasing thrombin generation during trauma-related bleeding include FFP, prothrombin complex concentrate (PCC) and activated recombinant factor VII (rFVIIa) [8,37,85-87]. rVIIa was studied in two randomized controlled trials and failed to show a survival benefit [85,88].

Limited data on the use of PCC in trauma are currently available. In one study, administration of fibrinogen concentrate (n = 128), along with PCC (n = 98), in trauma patients (n = 131) produced more favourable survival rates than those predicted by the trauma injury severity score (TRISS) or revised injury severity classification (RISC) score [8]. Schöchl et al. also reported significantly higher transfusion rates for RBC and PC among trauma patients receiving haemostatic therapy with allogeneic blood products only, compared with those receiving coagulation factor concentrates [86]. However, no randomized controlled trials have been conducted to investigate this concept.

Robust safety data are lacking for the use of PCCs in the treatment of TIC. PCC is a procoagulant drug, and a possible risk of thromboembolic adverse events must be considered alongside patients' possible tendency towards thrombosis following trauma [79,89-91]. The risk of haemostatic imbalance relating to the prothrombotic nature of PCC therapy may be offset by the use of viscoelastic coagulation monitoring (EXTEM CT) to minimize the risk of excessive dosing. Although some PCCs contain the anticoagulant proteins C, S and Z, the main antagonist of factor II (antithrombin, ATIII) is either absent from PCC or present in far smaller quantities than would be needed to balance the procoagulant potential of factor II [92]. In addition, the administered quantity of factor II may be higher than that suggested by the labeled dose, because PCCs are standardized according to factor IX (e.g. in one product investigated by Kalina *et al*., the ratio of FII:FIX was 1.6) [92]. Co-administration of ATIII with PCC may be an option, particularly in patients with a lack of coagulation inhibitors. When time is available (e.g. oozing bleeding, in either the operating room or the ICU), the plasma ATIII level should be measured before PCC administration. However, clinical trial data are needed to confirm best practice regarding potential ATIII co-administration.

AUVA TRAUMA HOSPITAL TREATMENT ALGORITHM FOR TIC

Prolongation of EXTEM CT > 80 seconds

1. Rule out the following reasons for CT prolongation:

fibrinolysis (APTEM CT < EXTEM CT [not well established for assessing fibrinolysis])

heparin effect (HEPTEM CT < INTEM CT [e.g. following transfusion of cell- saver blood].

2. EXTEM CT > 80 seconds serves as a surrogate parameter for insufficient thrombin generation. PCC is a treatment option for improving thrombin production.

### Improvement of clot stability

Hyperfibrinolysis is associated with high mortality and increased risk of MT [13-15]. Kashuk et al. reported that even a small reduction (1 unit) in TEG clot strength (G value) 1 hour post-injury increased mortality by > 10%, and that primary fibrinolysis occurs in a higher percentage of patients requiring MT than in the overall group of trauma patients (34% vs. 18%) [13].

These observations are in line with data from the CRASH-2 study which showed an improved survival rate in patients receiving early antifibrinolytic therapy (tranexamic acid [TXA] 1 g over 10 minutes followed by an infusion of 1 g over 8 hours). Mortality in the placebo group was 16%, whereas in the TXA group it was 14.5% [87]. Surprisingly, mortality was increased among patients receiving TXA later than 3 hours post-trauma [93].

AUVA TRAUMA HOSPITAL TREATMENT ALGORITHM FOR TIC

TXA (15-20 mg per kg bodyweight) should be administered to all major trauma patients (ISS > 16), to all trauma patients admitted in shock, and to all trauma patients with hyperfibrinolysis confirmed by ROTEM test results (any major improvement in APTEM parameters compared to corresponding EXTEM parameters).

**Figure 3:**
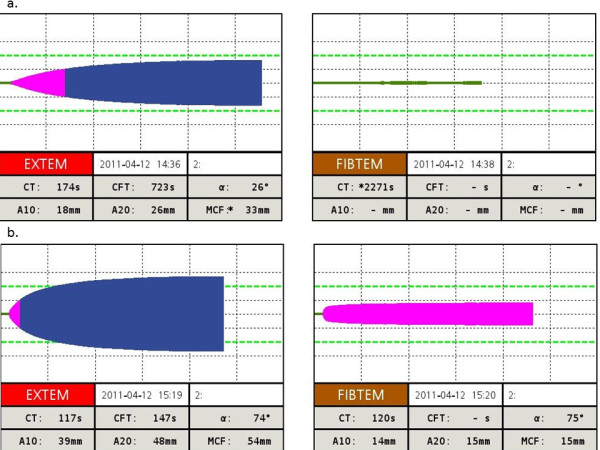
**ROTEM traces from a trauma patient treated according to the AUVA Trauma Hospital algorithm: **a. upon admission to the ER (EXTEM coagulation time and clot formation time are prolonged; maximum clot firmness is reduced; no clot formation in the FIBTEM test). b. 40 minutes after treatment with 2 g tranexamic acid, 10 g fibrinogen concentrate, 1800 U prothrombin complex concentrate and 1250 U factor XIII (normal coagulation).

Figure 2 depicts the "theragnostic" algorithm established in the AUVA Trauma Hospital, Salzburg, and Figure 3 displays an example of a severe bleeding patient treated according to this algorithm. The algorithm is not currently supported by data from randomized controlled trials, but clinical evidence has indicated its viability in terms of safety and effectiveness [8,86]. Because of the poor evidence base and international variations in the approval status of coagulation factor concentrates, it would be inappropriate to advocate widespread adoption at this stage. However, the lack of well-designed randomized controlled trials supporting the use of allogeneic blood products must also be considered [94], together with the empirical advantages of individualized, goal-directed therapy.

## Conclusion

The "theragnostic" concept is based on rapidly available whole-blood viscoelastic test results. Haemostatic therapy is individualized according to the patient's actual needs. Compared with predefined ratio-driven approaches to administering allogeneic blood products, individualized coagulation management potentially reduces the risks of both under-transfusion (increased risk of bleeding) and over-transfusion (increased risk of ARDS, acute lung injury, sepsis and MOF). Coagulation factor concentrates offer possible advantages over FFP, but the potential risks must be considered carefully. To confirm the efficacy and safety of individualized haemostatic management based on coagulation viscoelastic tests, randomized controlled studies are mandatory.

## Abbreviations

aPTT: activated partial thromboplastin time; ALI: acute lung injury; ARDS: acute respiratory distress syndrome; CA10: clot amplitude after 10 minutes' running time; CT: coagulation time; CFT: clot formation time; CP: cryoprecipitate; ER: emergency room; FFP: fresh-frozen plasma; HF: hyperfibrinolysis; ICU: intensive care unit; k-time: kinetic time; MA: maximum amplitude; MCF: maximum clot firmness; MOF: multiple organ failure; INR: international normalized ratio; MT: massive transfusion; MTP: massive transfusion protocol; PC: platelet concentrate; PCC: prothrombin complex concentrate; POC: point of care; PT: prothrombin time; PTI: prothrombin time index; RBC: red blood cells; r-time: reaction time; ROTEM: rotational thromboelastometry; TRALI: transfusion-related lung injury; TEG: thrombelastography; TIC: trauma-induced coagulopathy; TXA: tranexamic acid; U: units

## Competing interests

HS, CS, MM and KG received speakers' fee from CSL Behring and TEM international. HS and CS received research grants from CSL Behring. TEM international provided reagents and devices for studies. WV declares no competing interest.

## Authors' contributions

HS wrote the initial draft of the manuscript and performed the literature research. WV, MM, CS and KG edited and reworded parts of the manuscript. All authors read and approved the final manuscript.
